# Novel genomic resources for a climate change sensitive mammal: characterization of the American pika transcriptome

**DOI:** 10.1186/1471-2164-14-311

**Published:** 2013-05-10

**Authors:** Matthew A Lemay, Philippe Henry, Clayton T Lamb, Kelsey M Robson, Michael A Russello

**Affiliations:** 1 Department of Biology, University of British Columbia, Okanagan Campus 3333 University Way, Kelowna, BC V1V 1V7, Canada; 2 Present address: Ecosystem Science and Management Program, University of Northern British Columbia, 3333 University Way, Prince George, BC V2N 4Z9, Canada

**Keywords:** Adaptation, Elevation gradient, Next-generation sequencing, *Ochotona princeps*, Population genomics, Single nucleotide polymorphisms

## Abstract

**Background:**

When faced with climate change, species must either shift their home range or adapt *in situ* in order to maintain optimal physiological balance with their environment. The American pika (*Ochotona princeps*) is a small alpine mammal with limited dispersal capacity and low tolerance for thermal stress. As a result, pikas have become an important system for examining biotic responses to changing climatic conditions. Previous research using amplified fragment length polymorphisms (AFLPs) has revealed evidence for environmental-mediated selection in *O*. *princeps* populations distributed along elevation gradients, yet the anonymity of AFLP loci and lack of available genomic resources precluded the identification of associated gene regions. Here, we harnessed next-generation sequencing technology in order to characterize the American pika transcriptome and identify a large suite of single nucleotide polymorphisms (SNPs), which can be used to elucidate elevation- and site-specific patterns of sequence variation.

**Results:**

We constructed pooled cDNA libraries of *O*. *princeps* from high (1400m) and low (300m) elevation sites along a previously established transect in British Columbia. Transcriptome sequencing using the Roche 454 GS FLX titanium platform generated 780 million base pairs of data, which were assembled into 7,325 high coverage contigs. These contigs were used to identify 24,261 novel SNP loci. Using high resolution melt analysis, we developed 17 of these SNPs into genotyping assays, which were validated with independent DNA samples from British Columbia Canada and Oregon State USA. In addition, we detected haplotypes in the NADH dehydrogenase subunit 5 of the mitochondrial genome that were fixed and different among elevations, suggesting that this may be an informative target gene for studying the role of cellular respiration in local adaptation. We also identified contigs that were unique to each elevation, including a high elevation-specific contig that was a positive match with the hemoglobin alpha chain from the plateau pika, a species restricted to high elevation steppes in Asia. Elevation-specific contigs may represent candidate regions subject to differential levels of gene expression along this elevation gradient.

**Conclusions:**

To our knowledge, this is the first broad-scale, transcriptome-level study conducted within the Ochotonidae, providing novel genomic resources for studying pika ecology, behaviour and population history.

## Background

When faced with rapidly changing climates, many species are expected to undergo widespread shifts in their distribution in order to maintain optimal physiological balance with their environment [1]. However, for species with fragmented habitats and those with limited dispersal capacities, range shifts may not be a viable option, and rapid adaptation may represent the only alternative to local extinction [2,3]. In order to predict the ability of these species to evolve *in situ* to changing environmental conditions, studies examining local adaptation along elevation gradients have emerged as model systems to predict the impact of climate change on species persistence and survival [4].

The American pika (*Ochotona princeps*) is a small alpine lagomorph with a discontinuous distribution throughout the mountain ranges of western North America [3,5]. Pikas are typically restricted to high-elevation talus slope ecosystems, which provide close proximity to meadows for foraging and a complex habitat for behavioural thermoregulation [6,7]. American pikas likely originated from an Asian ancestor that arrived in North America via the Bering land bridge [8]. During the warming that followed the Wisonsinan glaciation, paleontological evidence suggests that the distribution of *O*. *princeps* contracted northward and to higher elevations [9], effectively stranding extant populations on high-elevation ‘habitat islands’. Currently, the lower limits of *O*. *princeps* populations are constrained by an inability to tolerate thermal stress, while their high elevation distribution is enabled by adaptation to hypoxic environments [10]. The uniquely fragmented nature of their habitat has propelled *O*. *princeps* to a focal mammalian species for more general studies of metapopulation dynamics, island biogeography, and source-sink dynamics [9,11].

Pikas have also emerged as an important study species for investigating extinction risk in the face of rapidly changing climates [5,7,12-15]. Unlike the majority of woodland montane fauna whose continuous habitat allows for cross-valley dispersal among mountain ranges, pikas reliance on high-elevation talus habitat precludes their ability for dispersal to cooler latitudes [9]. Instead, it is hypothesized that the continued persistence of pikas will depend on *in situ* adaptation to changing climatic conditions, leading some to suggest that they may become the first mammalian species to go extinct due to the direct effects of climate change [16]. Investigating the genetic basis of adaptation in pikas may provide insight into the underlying mechanisms by which contemporary evolution occurs in response to rapidly changing environments. However, this research is hindered by a lack of available genomic resources. For example, a recent genome scan using amplified fragment length polymorphisms (AFLPs) among populations continuously distributed along three elevation gradients (0 m-1500 m) identified 15 outlier loci (out of 1509) putatively exhibiting signatures of divergent selection associated with summer mean maximum temperature and precipitation (Philippe Henry and Michael Russello, unpublished data). Yet, the anonymity of AFLP loci precluded the identification of underlying genomic regions associated with these candidate loci.

The rise of next-generation sequencing technologies provides tools for rapidly generating DNA sequence data for non-model organisms that have previously lacked genomic resources. When combined with statistical population genomics approaches [17], these data can be used to test for signatures of natural selection in wild populations and identify candidate gene regions associated with local adaptation [18]. Single nucleotide polymorphisms (SNPs) have emerged as the marker of choice for population-level genotyping in the genomics era [19,20]. Due to their high coverage across the genome, ease of genotyping, and direct relationship with underlying gene function, SNPs represent an improvement over conventional markers such as AFLPs and microsatellites for identifying genome-wide patterns of adaptive genetic variation [21]. Despite their utility for population level studies, large-scale SNP resources are still lacking for many species, including *O*. *princeps*.

The purpose of this study was to harness next-generation sequencing technology in order to elucidate elevation-specific patterns of sequence variation in *O*. *princeps*. We generated transcriptome-wide sequence data for pooled cDNA libraries from high (1400 m) and low (300 m) elevation sites along a previously established elevation gradient in the British Columbia (BC) Coast Mountains [13]. The resulting high coverage contigs and large suite of SNP loci represent novel genomic resources for studying pika ecology, behaviour and population history, and enable direct investigations of potential biotic responses to changing environments.

## Results and Discussion

### Sequencing and assembly

Using the Roche 454 GS FLX titanium platform, we generated ~780 million bases of transcriptome sequence data corresponding to 1.6 × 10^6^ and 1.5 × 10^6^ reads for the high and low elevation cDNA libraries, respectively (Table 1).

**Table 1:** **Summary of the next**-**generation sequence data obtained from each cDNA library**

	**High elevation**	**Low elevation**
No. of bases	424,134,294	357,655,427
No. of reads	1,589,727	1,455,497
Mean read length	266.8	245.7

**Figure 1:**
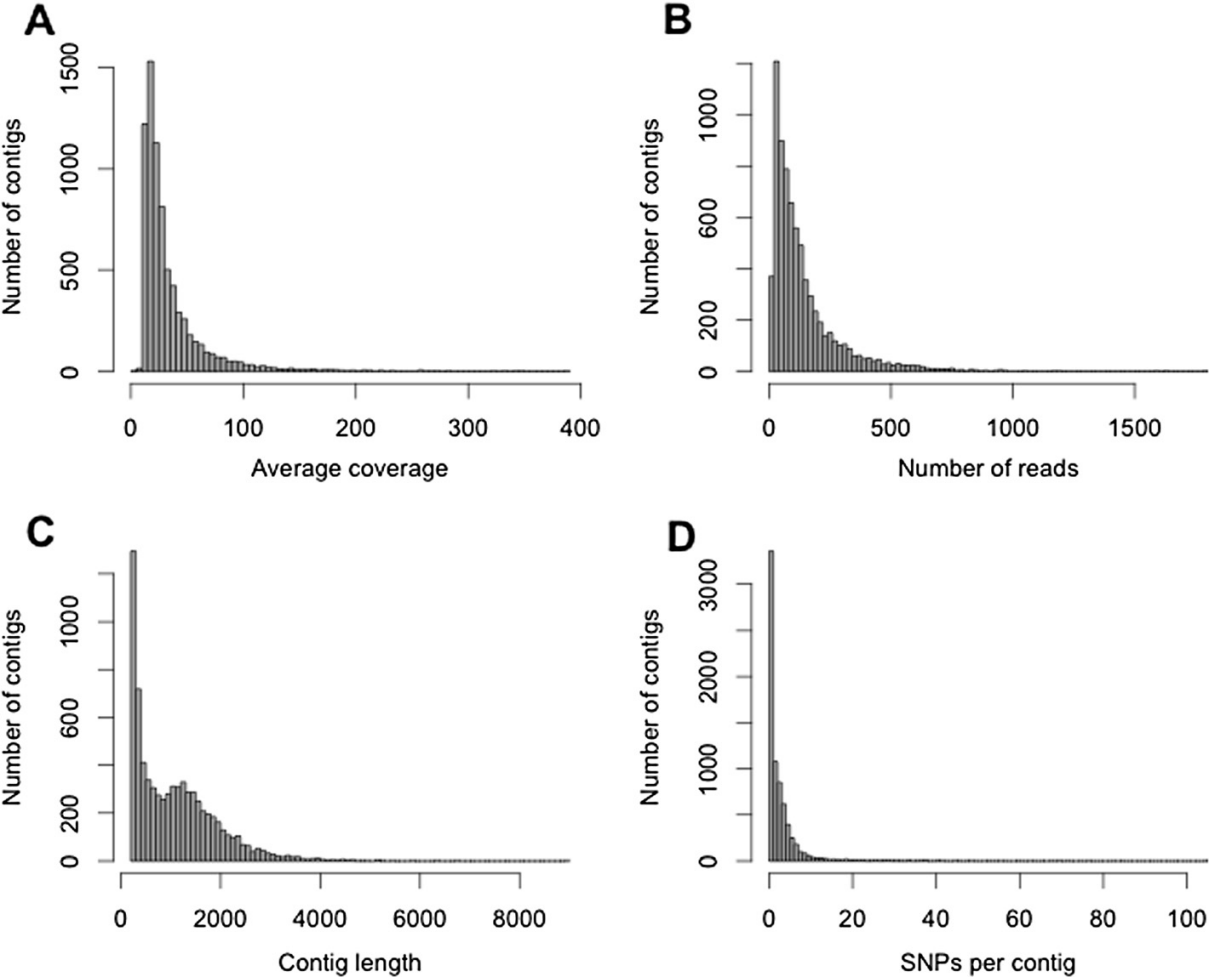
**Characterization of contigs present in the high coverage dataset.** Histograms represent (**A**) average coverage of each contig (mean = 33×), (**B**) number of reads that mapped to each contig (mean = 137.0), (**C**) contig lengths (mean = 1079.5 base pairs), and (**D**) the number of SNPs for each of the high coverage contigs.

A *de novo* assembly was first carried out using the trimmed reads from both elevations in order to generate reference contigs; this assembly incorporated 66% of the transcriptome reads to produce 102,175 contigs. We then mapped the raw reads back to these reference contigs separately for each elevation in order to generate a refined dataset consisting only of contigs that had a minimum average coverage of 5× for each elevation and a minimum length of 200 bases. The resulting dataset (hereafter referred to as the high coverage dataset) consisted of 7,325 contigs with a mean coverage of 33 reads per site (Table 2; Figure 1; Additional files 1 and 2). These contigs represent less than 1% of the *O*. *princeps* genome, which initial low coverage estimates indicate is 1.92 Gb in length [22].

**Table 2:** **Summary of the contigs present in each *O***. ***princeps *dataset**

	**Total contigs**	**High coverage dataset**^**1**^	**High elevation unique contigs**^**2**^	**Low elevation unique contigs**^**2**^
No. of contigs	102,175	7,325	1,038	304
Mean coverage	5.5	33.2	7.4	8.0
Mean length	534.6	1,079.5	383.2	354.1
Mean no. of reads	16.6	137.0	11.4	12.9

^1^ In the high coverage dataset each contig has a minimum length of 200 bases and a minimum of 5× coverage for each ecotype.

^2^ Contigs composed of reads from a single elevation. Minimum length = 200 bases; minimum coverage = 5×.

We performed an additional *de novo* assembly (similarity = 0.90) of the high coverage contigs in order to identify sequences that either partially or totally overlapped. This assembly revealed some redundancy in the contig dataset. Out of the 7,325 contigs in the high coverage dataset, 588 contigs (8.0%) aligned with one other contig, and 221 contigs (3.0%) aligned with two or more other contigs. The remaining 6,516 contigs (89.0%) were unique and did not show similarity with any other contig.

### Transcriptome annotation

**Figure 2:**
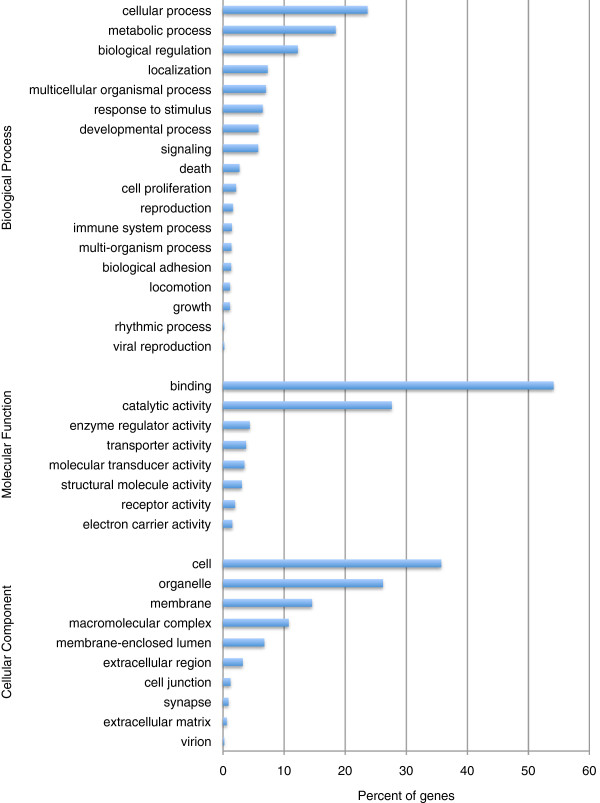
**Functional annotation of contigs in the high coverage dataset.** The distribution of gene ontology (GO) terms is given for each of each of the three main GO categories (biological process, molecular function, and cellular component).

A BLAST search of all contigs in the high coverage dataset (7,325 sequences) produced 3,788 positive hits (BLASTx search of the NCBI nr database, minimum e-value cut off = 10^-6^; average e-value = 3.4 × 10^-9^; Additional file 2). Of the positive BLAST hits, only 14 were matches to sequences from *Ochotona sp*., highlighting the current lack of genomic resources available for pikas; 1,215 contigs had positive matches to published genes from the European rabbit (*Oryctolagus cuniculus*), which is the closest model organism to *O*. *princeps*. Of the contig sequences with positive BLAST match, 2,279 were subsequently annotated with one or more gene ontology (GO) terms (Figure 2).

### SNP detection

Among the high coverage contigs (n = 7,325), 5,357 had SNPs that fell within our detection parameters (Additional file 3). The total number of SNPs identified was 24,261, of which 3,399 were polymorphic among pika from both elevations, 10,504 were polymorphic in low elevation but fixed in high elevation pika, and 10,269 were polymorphic in the high elevation but fixed in low elevation. There were 89 SNPs within our detection parameters that appeared to be fixed for alternate alleles in the two elevations. The ratio of transitions to transversions was 3.86, and the difference in the frequency of the major allele between the two elevations ranged from <1 to 100% (mean divergence = 21%).

Among these data, the frequency of SNPs that appear to be fixed at one elevation may be artificially inflated due to the small sample size (n = 3 for each elevation) used to generate the transcriptome sequences. There is a high probability that low frequency alleles would not have been present among the individuals sampled. In addition, the SNP detection parameters required a minimum coverage of eight reads at a polymorphic site to be included in the data. If the samples from one elevation had low coverage at a particular site, it would appear to be fixed even if there was variation present. These potential biases reflect the trade-off between avoiding false SNPs resulting from sequencing error, while attempting to account for all possible variation in the data.

### SNP validation

Primer pairs were designed for 85 SNP loci such that they amplified an ~200 base pair fragment that contained a single SNP (Additional file 4). Of these loci, 26 had successful PCR amplification, were free of introns, and produced sufficiently clear high resolution melt (HRM) signal to attempt the subsequent genotyping validation. High resolution melt analysis was then used to genotype 10 high and 11 low elevation *O*. *princeps* from the Bella Coola, BC study site as well as 21 samples collected at an independent location in the Columbia River Gorge, Oregon, USA.

Sanger re-sequencing of representative samples from each melt curve obtained from these 26 loci was used to assign genotypes to each cluster. From the panel of 26 SNPs for which Sanger validation was attempted, 17 loci (65%) yielded evidence of consistently scorable nucleotide polymorphism. Sanger sequence data for the remaining nine loci confirmed that the expected SNP site was indeed polymorphic, however, the resulting HRM curves were not sufficiently discrete to enable accurate genotype assignment (i.e. Sanger sequencing revealed that multiple melt curves had the same genotype or identified multiple genotypes within the same cluster). Given that in these cases Sanger sequencing confirmed the presence of the expected polymorphism, we conclude that the failed assays were not due to errors with the initial SNP detection but rather reflect the limitations of the HRM assays at those loci. For example, the presence of additional polymorphic sites within the amplicon [23] and loci containing Class 3 (C/G) or Class 4 (A/T) SNPs [24] may result in complicated or weakly differentiated clusters unsuitable for HRM genotyping.

Eight of these 17 retained SNP loci exhibited sequence similarity to structural or regulatory genes in the NCBI database (*Ocp4162*, *Ocp6361*, *Ocp6774*, *Ocp7498*, *Ocp*14764, *Ocp*15508, *Ocp*17339, *Ocp*102175; Additional file 2). We found no evidence of linkage disequilibrium among any of the loci that were successfully typed in our samples. Four of 17 loci showed a significant deviation from Hardy-Weinberg equilibrium (HWE), however each instance was restricted to a single elevation at one location (Table 3).

**Table 3:** Genetic diversity estimates from loci that were successfully genotyped using HRM analysis

**Locus**	**H**_**O**_**/H**_**E**_			
	**BC high**	**BC low**	**Oregon high**	**Oregon low**
*Ocp687*	0.33/0.28	0.25/0.47	**0**.**00**/**0**.**00**	**0**.**00**/**0**.**00**
*Ocp2098*	**0**.**00**/**0**.**00**	0.50/0.50	**0**.**00**/**0**.**00**	**0**.**00**/**0**.**00**
*Ocp4162*	0.29/0.49	**0**.**00**/**0**.**00**	**0**.**00**/**0**.**00**	0.00/0.50*
*Ocp4280*	**0**.**00**/**0**.**00**	1.00/0.50*	0.22/0.35	0.33/0.44
*Ocp4649*^a^	0.00/0.44	0.25/0.22	0.33/0.28	0.00/0.44
*Ocp5210*	0.67/0.44	0.75/0.47	**0**.**00**/**0**.**00**	0.00/0.49*
*Ocp6361*	**0**.**00**/**0**.**00**	0.00/0.38*	**0**.**00**/**0**.**00**	0.00/0.44
*Ocp6774*	1.00/0.50	**0**.**00**/**0**.**00**	**0**.**00**/**0**.**00**	0.00/0.28
*Ocp7498*	0.67/0.44	**0**.**00**/**0**.**00**	0.43/0.34	0.60/0.42
*Ocp8183*	0.00/0.44	0.75/0.47	**0**.**00**/**0**.**00**	**0**.**00**/**0**.**00**
*Ocp8469*	0.33/0.28	0.25/0.22	0.00/0.24	**0**.**00**/**0**.**00**
*Ocp14764*	0.67/0.44	**0**.**00**/**0**.**00**	0.43/0.34	0.29/0.24
*Ocp15503*	0.67/0.44	**0**.**00**/**0**.**00**	**0**.**00**/**0**.**00**	**0**.**00**/**0**.**00**
*Ocp15508*	0.11/0.28	0.11/0.10	**0**.**00**/**0**.**00**	**0**.**00**/**0**.**00**
*Ocp17339*	**0**.**00**/**0**.**00**	0.25/0.47	0.43/0.34	0.29/0.41
*Ocp102174*	0.11/0.28	0.30/0.46	**0**.**00**/**0**.**00**	**0**.**00**/**0**.**00**
*Ocp102175*	0.29/0.41	0.11/0.28	0.33/0.50	**0**.**00**/**0**.**00**

* Significant deviation from HWE; bold denotes that the locus was monomorphic in that population.

^a^ X-linked locus.

All 17 loci tested were polymorphic among the 21 DNA samples from BC. Four of these loci were fixed for a single allele at high elevation and five loci were fixed for a single allele at low elevation (Table 3), potentially indicating elevation-specific patterns in the distribution of genetic variation. The remaining eight loci were polymorphic at both elevations in BC.

Among the DNA samples from Oregon, six loci were monomorphic. Of the remaining 11 loci, four were fixed for a single allele at the high elevation and two were fixed for a single allele at the low elevation site. Reduced genetic variation in samples from Oregon is likely representative of ascertainment bias (and low sample sizes), given that transcriptome sequencing and initial SNP discovery utilized tissue samples from BC.

### Mitochondrial DNA

There was a high coverage of reads across all genes in the *O*. *princeps* mitochondrial genome [GenBank: AJ537415], with 11,040 trimmed reads (0.4%) aligning to the published reference sequence. In addition, a BLASTx search of the high coverage dataset revealed 103 contigs that associated with the mitochondria.

**Figure 3:**
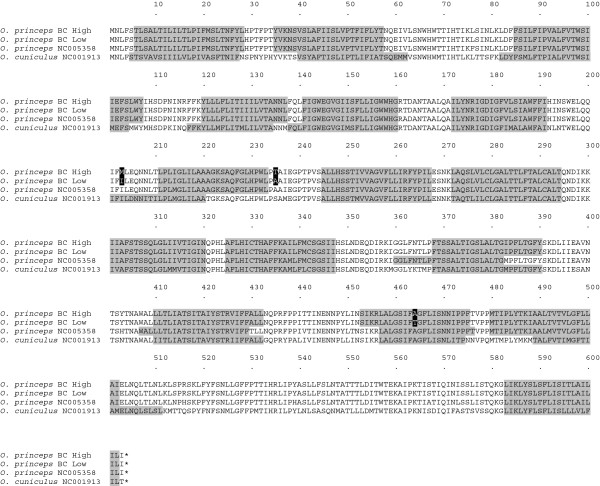
**Sequence alignment and secondary structure prediction of the ND5 gene in *O. princeps *haplotypes.** The first two rows in the sequence alignment are from *O*. *princeps* sampled at high and low elevations in BC, respectively. The second two rows are the homologous sections from the published mitochondrial genomes of *O*. *princeps* [GenBank: NC005358] and *Oryctolagus cuniculus* [GenBank: NC001913]. Amino acids in white bold and black background indicate non-synonymous substitutions fixed at low and high elevation pikas in BC. Predicted transmembrane domains are shaded in gray. For the BC samples, residues 16–266 and 405–551 are the result of Sanger sequencing four individuals per elevation; the remaining residues are inferred from transcriptome read data of three individuals per elevation.

Of particular note, we detected multiple SNPs within two contigs (contigs 1829 and 24554) that sequence-similarity searches revealed corresponded to portions of the NADH dehydrogenase subunit 5 (ND5) region of the mitochondrial genome. Two distinct haplotypes were detected across a total of eight polymorphic sites that associated with elevation in BC (Figure 3). Three of these polymorphic sites were non-synonymous substitutions, two of which occurred in loop regions, while a third was found within a predicted transmembrane domain.

NADH dehydrogenase is the first and largest enzyme complex in the respiratory chain of the oxidative phosphorylation machinery, and plays a central role in energy metabolism [25,26]. A broad-scale study of adaptive evolution of the mitochondrial genome of 41 placental mammals revealed signatures of positive selection in the NADH dehydrogenase complex, largely restricted to the loop regions of the proton pumps, including ND5 [26]. Additional studies [27-29] have also detected positively selected sites in ND5, with adaptive changes in the piston arm suggested to have influenced fitness during the evolution of Pacific salmon species [29]. Future studies utilizing population level samples spanning the entire elevation gradient in BC are required to further investigate the role of ND5 in local adaptation of *O*. *princeps* across varying environments.

### Contigs unique to each elevation

Additional datasets were generated containing contigs that were only composed of transcriptome reads from either the high or low elevation (Table 2; Additional file 5). BLAST searches (BLASTx, NCBI nr database, max e-value = 10^-06^) of these elevation-specific contigs produced 88 positive matches in the high elevation dataset (mean e-value = 1.8 × 10^-08^) and 83 positive hits among contigs unique to low elevation (mean e-value = 1.0 × 10^-08^).

Interestingly, there was a high-elevation-specific contig (contig 31687; Additional file 5) that was a strong match with the hemoglobin alpha chain from high elevation samples of both the Chinese red pika [*O*. *erythrotis*, GeneBank: JX827174, e-value = 1.1 × 10^-56^] and the plateau pika [*O*. *curzoniae*, GenBank: EF429202, e-value = 1.2 × 10^-55^], species restricted to high elevation steppes in Asia (3000-5000m; [30]). An additional assembly of raw reads to both the hemoglobin reference sequence [EF429202] and to the associated contig (contig 31687) confirmed that low elevation reads were indeed absent, rather than being misassembled during the initial *de novo* assembly and read-mapping (CLC Genomics Workbench v.5.5, similarity 0.9, length fraction 0.5; data not shown).

Hemoglobin is a key component of oxygen storage and regulation, and plays an important role in physiological adaptation to different environments [31]. A host of studies have demonstrated an association of hemoglobin alpha chain haplotype frequency with elevation in mammals [31-34]. Here, hemoglobin alpha chain transcripts were only detected among the high elevation sequencing reads. We Sanger sequenced the hemoglobin alpha chain in our DNA samples of *O*. *princeps*, revealing no variation at the nucleotide level within or among elevations (data not shown). This result may be indicative of differential gene expression across elevations, with expression among the low elevation samples occurring below our detection level, even after the normalization of transcripts. Additional studies are required to further elucidate the role of hemoglobin alpha chain, if any, in local adaptation of *O*. *princeps*.

Gradients in latitude and elevation can been useful for predicting the impact of climate change on natural populations. For example, in the case of mountain species, low-elevation populations may possess unique genetic variation associated with adaptation to higher temperature; if present, such adaptations might provide insight into the ability of high elevation populations to adapt in response to climate change. While our study was not designed to test predictions related to climate change, we provide novel sequence data from genes expressed by *O*. *princeps* at both low and high elevations, which provides a valuable resource for future research.

## Conclusions

To our knowledge, this is the first broad-scale, transcriptome-level study conducted within the Ochotonidae, providing novel genomic resources to inform studies of pika ecology, behaviour, and population history, while enabling direct investigations of potential biotic responses to changing environments. We identified 24,261 novel SNPs among *O*. *princeps* inhabiting different elevations. We detected SNPs and haplotypes that were fixed and different among elevations, and identified the ND5 region of the mitochondrial genome as a promising target gene for further studying the role of cellular respiration in local adaptation to varying environments. We also found contigs that were unique to each elevation, including hemoglobin alpha chain, which may represent candidate regions subject to differential gene expression along this elevation gradient. Although this RNAseq approach was successful at identifying a large number of novel SNP loci, information on allele frequencies was limited by the small number of individuals used in the pooled libraries. Emerging protocols that utilize combinatorial labelling methods and Restriction Associated DNA (RAD; [35,36]) sequencing may provide more efficient and cost-effective alternatives for simultaneously discovering SNPs in non-model organisms and genotyping population-level samplings.

## Methods

### Sample collection, RNA extraction, and next generation sequencing

Sample collection was carried out in Tweedsmuir South Provincial Park in the Bella Coola Valley, BC, Canada, which is a mountainous region with talus slopes scattered throughout. Previous work in Tweedsmuir Park has characterized neutral and adaptive genetic variation in *O*. *princeps* along three elevational transects [13]. Tissue collection in the current study focussed on ‘The Hill’ site, which has an elevational cline from 301 m (low elevation site) to 1433m (high elevation site) above sea level. A recent study demonstrated an average temperature difference of up to six degrees between low and high elevation sites in summer [37], which is of a similar magnitude to predicted temperature shifts for this part of the world during the next century. Three individuals at the low elevation site and three individuals at the high elevation site were collected using Tomahawk Live traps and sacrificed in the field. Sample collection was carried out in accordance with University of British Columbia Animal Care Certificate #A07-0126 and sampling permits from the BC Ministries of Environment (# 78470–25) and Forests, Lands and Natural Resource Operations (NA11-69259). Five tissue types (brain, gonad, heart, liver, lung) from each individual were immediately harvested and placed in separate 5 ml screw-cap vials containing 2.5 ml of RNALater solution. Samples were held at 4°C for 24 hrs and then stored at −20°C until needed. RNA was extracted from each tissue using the RNEasy Universal MiniKit (Qiagen) following the manufacturer’s protocol. All specimens were accessioned within the mammal collection at the Royal British Columbia Museum (RBCM catalogue numbers 20919–20924; Additional file 6).

Two normalized cDNA libraries (Evrogen, Russia) were constructed using pooled RNA from all high elevation (5 tissues × 3 individuals) and low elevation (5 tissues × 3 individuals) samples. The two resulting cDNA libraries were each subject to a full run of 454 GS FLX Titanium sequencing at the Genome Quebec core facility. Pooling of multiple individuals in each sample was used to provide a preliminary indication of the genetic variation within and among elevations; the combination of five tissue types for each individual was used to maximize the diversity of expressed genes present in each library. RNA samples were normalized in order to increase the detection of rare transcripts in the sequence data.

### Assembly

Initial trimming of the read data was performed using the CLC Genomics Workbench (CLC Bio) v.4.8 such that very short reads (<100 bases), terminal nucleotides (five from each end), low quality reads (quality limit 0.05), and 454 sequencing adapters and primers were removed from the dataset. A *de novo* assembly using the CLC Genomics Workbench v. 5.1 was then carried out (similarity = 0.90) in order to generate reference contigs. To facilitate a comparison of sequence variation between the two elevations, the consensus sequence from each reference contig was used to map the high and low elevation reads separately (similarity = 0.90, length fraction 0.5). We retained only those contigs that had a minimum length of 200 bases and an average coverage greater than 5× for each elevation (hereafter referred to as the high coverage dataset).

We performed a *de novo* assembly (CLC Genomics Workbench v. 5.5; similarity = 0.90, length fraction 0.5) using all contigs in the high coverage dataset in order to identify contigs that partially overlapped. Redundancies among the contigs may be indicative of alternative splicing within the transcriptome data.

We also generated datasets containing those contigs that were composed of reads from only a single elevation (minimum coverage = 5×; minimum length = 200 bases). These two ‘elevation-unique’ datasets may suggest target genes for subsequent studies examining differences in gene expression among elevations. For all analyses, assembly and mapping parameters were optimized by comparing the results of multiple runs at different levels of similarity and length fraction.

### Transcriptome annotation

We conducted sequence similarity searches for the high coverage dataset (n = 7,325 contigs) using Blast2GO v.2 [38,39]. For these analyses, a BLASTx search was performed using the NCBI nr database (maximum e-value threshold = 10^-6^, HSP length cut-off = 33, top 5 hits were retained). In addition, gene ontology (GO) analysis was carried out, which provides hierarchically structured information with respect to molecular function, biological process, and cellular component. Annotations were assigned using Blast2GO (maximum e-value threshold = 10^-6^, HSP length cut-off = 20, GO weight 5). In addition, a BLAST search (BLASTx; same parameters as above) was carried out for all contigs in each of the two elevation-unique datasets.

### SNP discovery

The working dataset of high coverage contigs was screened for SNPs using the CLC Genomics Workbench v. 5.5 (minimum coverage 8×, minimum variant frequency 10%, minimum number of reads per allele = 2, minimum central quality 20). SNP detection was carried out separately for the two elevations and the resulting SNP tables were combined so that each site could be characterized as either: (a) polymorphic in both elevations; (b) fixed in one elevation, polymorphic in the other; or (c) fixed for different alleles in each elevation. In order to putatively identify the sites of greatest differentiation between elevations, a divergence value based on the index implemented in Juekens et al. [40] was calculated for each SNP, defined as the absolute value of the difference in the frequency of the major allele among elevations.

### SNP validation

A panel of SNPs with divergence values ≥50% was used to genotype an independent sample of *O*. *princeps* in order to test this ascertainment procedure. Validation of candidate SNPs was carried out following a pipeline similar to that implemented by Seeb et al. [23]. Briefly, primers were designed using Primer3[41] such that they would amplify an ~200bp fragment that encompassed a single SNP (Additional file 4). An initial PCR was used to identify loci that produced a single clean product of the anticipated size; these loci were then used to genotype 42 individuals using High Resolution Melt (HRM) analysis (see below).

Each test PCR contained 1.25 μl of 10× buffer, 1.25 μl of 2 mM dNTP mix (Kapa Biosystems), 1.0 μl of BSA, 0.5 μl of 10 mM forward and reverse primer, 0.5 units of *Taq* polymerase (AmpliTaq Gold, Applied Biosystems), 20–100 ng of DNA template, and ultra pure water for a total reaction volume of 12.5 μl. For each reaction, a touchdown PCR procedure was implemented using a Veriti thermal cycler (Applied Biosystems). The program had an initial denaturation at 95°C for 10 minutes, followed by 8 cycles at 95°C for 30 seconds, 59°C for 30 seconds, and 72°C for 30 seconds with the annealing temperature decreasing by 1.0°C per cycle. This was followed by 27 cycles at 94°C for 30 seconds, 51°C for 30 seconds, and 72°C for 30 seconds. The final cycle had an extension of 72°C for 10 minutes and was then held at 4°C. PCR products were run on a 1.5% agarose gel in order to obtain a preliminary assessment of the quality and size of the amplicon. Loci that failed to amplify, showed evidence for the presence of introns (larger products than expected), or had multiple bands were not retained for subsequent analyses.

High resolution melt analysis was carried out using DNA samples of *O*. *princeps* from both the Bella Coola Valley, BC, Canada and the Columbia River Gorge, Oregon, USA. Sampling procedures for the Bella Coola samples have been previously reported by Henry et al. [13]. We used DNA samples from the same high elevation (n = 10 individuals) and low elevation (n = 11 individuals) sites from which the tissue samples for the transcriptome sequencing were collected. Oregon samples were collected in the summer of 2012 from sites at both high (n = 11) and low elevations (n = 10) using non-invasive hair-snares [42]. DNA extraction was carried out using a DNA IQ™ Tissue and Hair Extraction Kit (Promega, Madison, WI, USA) kit following the protocol outlined in Henry et al. [13].

Each HRM reaction contained 7.2 μl of Precision Melt Supermix (BioRad), 0.4 μl of each primer, 20–100 ng of DNA template, and ultra pure water for a total reaction volume of 20 μl. High resolution melt analyses were run in 96 well plates on a BioRad CFX96 Touch™ real time PCR detection system. A two-step touchdown PCR protocol was used, starting with an initial denaturation step at 95°C for 2 minutes, followed by 9 cycles of 95°C for 10 seconds, 60°C for 30 seconds, with the annealing temperature decreasing by 1°C per cycle. This was followed by 43 cycles of 95°C for 10 seconds and 50°C for 30 seconds. The final PCR cycle consisted of 95°C for 30 seconds followed by 55°C for 1 minute. A plate read was obtained at the end of every PCR cycle. The melt curve data were collected starting at 70°C and increasing by 0.2°C every 10 seconds to a maximum of 95°C. A plate read was obtained at every 0.2°C increment. Melt curve data were analyzed using BioRad Precision Melt Analysis™ software.

Loci that successfully amplified, were free of introns, and produced well-resolved clusters of HRM curves (n = 26 loci) were subjected to Sanger re-sequencing on an ABI 3130XL Genetic Analyzer (Applied Biosystems). Given that each HRM cluster should represent a single SNP genotype, 2–3 individuals from each cluster were sequenced in order to determine the genotype of each cluster. For each locus that was successfully genotyped, we calculated the expected and observed heterozygosity values, tested for linkage disequilibrium, and tested for deviations from Hardy-Weinberg equilibrium using GenePop v.4 [43]. Type I error rates were corrected for multiple comparisons using the sequential Bonferroni procedure [44].

### Mitochondrial DNA

Mitochondrial genes may represent an important component of adaptation to different elevations in *O*. *princeps*[45]. To assess the prevalence and sequence variation of mitochondrial genes in the transcriptome data, we used the annotated mitochondrial genome for *O*. *princeps* [GenBank: NC005358] as a reference for read mapping (CLC Genomics Workbench v.5.5, similarity = 0.90, length fraction 0.5).

For ND5, Sanger sequences were obtained from four high and four low elevation pikas from BC using primers designed based on transcriptome sequence data from contig 1829 and contig 24554 (Additional file 4). A touchdown PCR protocol was used, with an initial denaturation at 95°C for 10 minutes, then 8 cycles at 95°C for 30 seconds, 59°C for 30 seconds, and 72°C for 2 minutes. This was followed by 32 cycles at 95°C for 30 seconds, 51°C for 30 seconds, and 72°C for 2 minutes. The final cycle had an extension of 72°C for 7 minutes and was then held at 4°C. PCR products were purified using Exo-Sap-It (USB Corporation) and sequenced on an ABI 3130XL Genetic Analyzer (Applied Biosystems). The resulting data were aligned with previously published ND5 sequences from *O*. *princeps* [GenBank: NC005358] and the European rabbit [*Oryctolagus cuniculus*; GenBank: NC001913]. Transmembrane helices were predicted from translated amino acid sequences using the hidden Markov model implemented in TMHMM v2.0 [46].

## Competing interests

The authors declare that they have no competing interests.

## Authors’ contributions

MR and ML designed the study. ML extracted the RNA, analyzed the data, and prepared the manuscript. PH collected samples, assisted with data analysis and helped draft the manuscript. CL and KR collected samples, and assisted with genotyping and Sanger sequencing. MR obtained funding, collected samples, analyzed the data and helped to draft the manuscript. All authors have read and approved the final manuscript.

## Supplementary Material

Additional file 1 **High coverage contig sequences.** Text file (.txt) in FASTA format containing the sequence of all high coverage contigs used for SNP detection (minimum length = 200 bases, minimum coverage = 5**×** for each ecotype). Click here for file

Additional file 2 **Characterization of high coverage contigs.** Excel file (.csv) listing the length, coverage, number of reads, and top BLASTx hit for each of the high coverage contigs. Click here for file

Additional file 3 **SNP information.** Excel file (.csv) characterizing the 24,261 SNPs identified in the high coverage dataset. Click here for file

Additional file 4 **SNP primers.** A table (.doc) containing the primer sequences for all loci for which HRM validation was attempted. Click here for file

Additional file 5 **Elevation unique contig sequences.** Text file (.txt) in FASTA format containing the sequence of all contigs that were composed entirely of reads from a single elevation (minimum length = 200 bases, minimum coverage = 5**×**). Click here for file

Additional file 6 **Sample collection.** Word document (.doc) describing the samples used to generate each cDNA library. Click here for file
