# Supplementary material for: Novel genomic resources for a climate change sensitive mammal: characterization of the American pika transcriptome
Source: BMC Genomics. 2013 May 10;14:311. doi: 10.1186/1471-2164-14-311 (PMC3662648; doi:10.1186/1471-2164-14-311)
Supplement: Additional file 4 — SNP primers. A table (.doc) containing the primer sequences for all loci for which HRM validation was attempted. [file 1471-2164-14-311-S4.doc]

| Locus Name | Contig | Position | Primers | Amplicon size |
| --- | --- | --- | --- | --- |
| *Ocp5145* | 5145 | 339 | F: ACACTGTTCAACCGGTTCCT | 211 |
|  |  |  | R: ACAGCCCACGCTTTTGTTAT |  |
| *Ocp18195* | 18195 | 198 | F: AGAGGTTGAAGGCCACAGG | 216 |
|  |  |  | R: CTCTGGGTGTGGCAGGAC |  |
| *Ocp8469* | 8469 | 197 | F: TTCTCTCTGTTGGATATACTCAAGTTC | 194 |
|  |  |  | R: AACTGGGGACAGCATTCTTCT |  |
| *Ocp18750* | 18750 | 187 | F: GTCCCTCTGCTCACTTACCC | 211 |
|  |  |  | R: TCATCAGATTTGTGGCCGTA |  |
| *Ocp1932* | 1932 | 497 | F: TGGAGAACTCCTTGGTCAGC | 188 |
|  |  |  | R: AAAGCAATGGTTGCAGGAAG |  |
| *Ocp27949* | 27949 | 160 | F: ATCGCTCCCACTATGCAAAA | 209 |
|  |  |  | R: TTTTCCACTACATCCCACCA |  |
| *Ocp3858* | 3858 | 288 | F: CCTCTGAAGCCGTACCAAAA | 208 |
|  |  |  | R: CCTTTAGGGAAAGGCAGGAC |  |
| *Ocp5492* | 5492 | 590 | F: TCAGGCACATTTCAGCAGTT | 213 |
|  |  |  | R: TCCTTGTCTCCTGGTAGTCCA |  |
| *Ocp39099* | 39099 | 118 | F: CCCACCAGACCTCACAAAAC | 209 |
|  |  |  | R: CCTGGGGCTTATGAAGACAA |  |
| *Ocp3096* | 3096 | 1020 | F: AAAAGGGATGAAGCCAGACA | 193 |
|  |  |  | R: CAACAGACACTCCCCTTTGG |  |
| *Ocp6774* | 6774 | 600 | F: TGCTTGGCTATGTGTTTTTCC | 218 |
|  |  |  | R: GGCTCCACAAGAATCGTCTC |  |
| *Ocp28089* | 28089 | 417 | F: TTTCAGGACGCTGTTTTTCA | 167 |
|  |  |  | R: ATGGCTTGCAACATCAGCTA |  |
| *Ocp18175* | 18175 | 181 | F: GCTTCTGACATAAGAATGGATTTC | 190 |
|  |  |  | R: TGTGCCCCCAAAGACAAG |  |
| *Ocp9701* | 9701 | 250 | F: GACACCTTTGTCCACGTCACT | 182 |
|  |  |  | R: GCCCGCAGTTTGATGTGTA |  |
| *Ocp18111* | 18111 | 334 | F: GCACTGGAGAGGGAGTGTGT | 220 |
|  |  |  | R: AGAAAACCCTGCCAGCAAC |  |
| *Ocp15468* | 15468 | 309 | F: TCACTGGAGGAAAACTCTGG | 212 |
|  |  |  | R: GAAAGCCAGTCAACTATTCTAGGG |  |
| *Ocp12228* | 12228 | 257 | F: CACGCAGAGTATAGGCTCCA | 192 |
|  |  |  | R: CTCTCTGGGAGGGATTCTGC |  |
| *Ocp550* | 550 | 161 | F: GTCATCACCCTCCTGCTGTG | 204 |
|  |  |  | R: CCACTGCTTCTCATGGTTCA |  |
| *Ocp3510* | 3510 | 1059 | F: TGGTGAAGGGAAACTCAGACA | 194 |
|  |  |  | R: GCCATGAGTTTAGGTTCAAACAG |  |
| *Ocp2992* | 2992 | 566 | F: ACCTTTCCCTGGGGCTCT | 167 |
|  |  |  | R: CCCAGCTGCGAGAGCAAG |  |
| *Ocp15503* | 15503 | 149 | F: CAGACATCAGTAAAACATCATATCCA | 199 |
|  |  |  | R: CTCCTGCTCTAGCGGTAAGG |  |
| *Ocp7498* | 7498 | 118 | F: GAGTATGCTTGCTCCCCAAC | 179 |
|  |  |  | R: GCAGGGCAAAAGGGAAAGTA |  |
| *Ocp6547* | 6547 | 1059 | F: AAAACTGCTTGAGACAGCTAACC | 174 |
|  |  |  | R: GGACACAGGAGCTACCCATC |  |
| *Ocp2483* | 2483 | 577 | F: CGGATGGGAATGATGTGAC | 199 |
|  |  |  | R: ATGCTGCAGGCTTCATCTTC |  |
| *Ocp20307* | 20307 | 249 | F: GACTGTCCAGGGGCTTCATA | 210 |
|  |  |  | R: CTTGCTTCCACTCTTGCTCA |  |
| *Ocp1474* | 1474 | 485 | F: CGCCAGAGAAGGTCTGACA | 220 |
|  |  |  | R: TTTCCTTCAGTGGTCCAGAA |  |
| *Ocp32163* | 32163 | 163 | F: GGTGTCTATTGAGTCGTGAAATAGAA | 150 |
|  |  |  | R: AGAAACCTTTTAAATGCAGCGAAT |  |
| *Ocp18936* | 18936 | 763 | F: GGGCACGAGTAGCTGAAGAG | 217 |
|  |  |  | R: CCAAGAGCATGTTCCAGAGA |  |
| *Ocp14216* | 14216 | 390 | F: GGCGTAGCGTTCACAAAGTT | 180 |
|  |  |  | R: GAGAGCATGGAATCCTATGAAA |  |
| *Ocp25410* | 25410 | 166 | F: TTGGTGTGCTGATAGGAACTG | 224 |
|  |  |  | R: TTGGACTCTAACCTTTTCTTTAGACC |  |
| *Ocp2280* | 2280 | 219 | F: ACAAAGTTACAAACCATGTTTCC | 179 |
|  |  |  | R: CAACTGCACTTCCAGTCACC |  |
| *Ocp1415* | 1415 | 1116 | F: GGAGGAGCTAACGGAAGAGAA | 205 |
|  |  |  | R: CGCACCTCCTTGATGTGAA |  |
| *Ocp738* | 738 | 204 | F: GACACCAGCTGGAACCTGAC | 150 |
|  |  |  | R: CATCTGCTCTTCTGTCCCTGT |  |
| *Ocp6417* | 6417 | 127 | F: TTGGCACGTATACATTGAAACA | 192 |
|  |  |  | R: CCTGGACTACCACCTGAACC |  |
| *Ocp2098* | 2098 | 896 | F: GCTTTTTGCAGAACAAAATTGA | 213 |
|  |  |  | R: TTCCTGCTTTTAATGTGTAGATATTTT |  |
| *Ocp1850* | 1850 | 442 | F: TTTTCTTTTTATGATAGGTCAGGACA | 211 |
|  |  |  | R: GGGTTGGCTTAAGTGTTTGG |  |
| *Ocp5843* | 5843 | 443 | F: GCCGAGGAAACTGAGACTTG | 217 |
|  |  |  | R: TCTCCTCTCTGTTGTTGCATAA |  |
| *Ocp4649* | 4649 | 1379 | F: TGAGGAGCAGAAATAAATGTTGG | 201 |
|  |  |  | R: CCCATCTCCTACTGAAGGACTC |  |
| *Ocp15942* | 15942 | 143 | F: CTGCCACCCTTAACCACAAA | 180 |
|  |  |  | R: GTTCGGTTTGTTGGCACTCT |  |
| *Ocp47360* | 47360 | 278 | F: CACCAACAGGCCGAGAAG | 212 |
|  |  |  | R: CCAAGTTTCACAGACTACCCAAA |  |
| *Ocp14764* | 14764 | 261 | F: TGGTTGGAGGAGGTTAGCTG | 160 |
|  |  |  | R: AGGTTTATTGGTCGGCTGCT |  |
| *Ocp1604* | 1604 | 644 | F: GGACACCTGTCCCTGTGG | 155 |
|  |  |  | R: GCACACGTGACCATCAAAGA |  |
| *Ocp18874* | 18874 | 875 | F: ATGGACACCAATGCCATTTA | 160 |
|  |  |  | R: AGTACACGTGGGCCCGTTC |  |
| *Ocp4280* | 4280 | 203 | F: TGCAACAGTGAAGAAATAGTACCC | 197 |
|  |  |  | R: AAGGGGTGGGTAACTTCACA |  |
| *Ocp9530* | 9530 | 587 | F: GCACTGCCGCTACTGATCTT | 179 |
|  |  |  | R: TCAATCCCATTTGGTCACAG |  |
| *Ocp5542* | 5542 | 473 | F: TTCCAAGGTCCCTTTGATTTT | 191 |
|  |  |  | R: TGGAAAAGTTTAGGCAAACCTG |  |
| *Ocp4162* | 4162 | 252 | F: GAGAGTCAGCACCTGGAGGA | 215 |
|  |  |  | R: TTCCGGATTTGTTCCTTGTC |  |
| *Ocp11432* | 11432 | 888 | F: CAAATTTCAAGGTGTATGATCTTGT | 185 |
|  |  |  | R: AGACACAAGAATGGTGGATGG |  |
| *Ocp2453* | 2453 | 1089 | F: ATCCCCTTTAATTTCCTTGC | 181 |
|  |  |  | R: AAAATGCAATCTCCTTTCAGAGTT |  |
| *Ocp13408* | 13408 | 274 | F: GCCACTCATGCCTTTCCTT | 151 |
|  |  |  | R: GTCCAGAATGCTCCAGGAAG |  |
| *Ocp8273* | 8273 | 919 | F: GCGGAAAGTCACAACATGAA | 152 |
|  |  |  | R: TCGGGTAACACTCCAGAACC |  |
| *Ocp5562* | 5562 | 702 | F: ATGGTGATTGGCCAGAAAAG | 192 |
|  |  |  | R: TGAGCCTTGGGTTAAACTGC |  |
| *Ocp17339* | 17339 | 970 | F: CAAATGTTTGAATTTGAAGTGGA | 148 |
|  |  |  | R: CACTAAGTGCCGACATCCAG |  |
| *Ocp8183* | 8183 | 1081 | F: TTGTAACAAGGCGAGCAGGT | 144 |
|  |  |  | R: CACTGCTGCAGCTTTCCAT |  |
| *Ocp21861* | 21861 | 553 | F: CGGGTAGATGCTGGTTCTGT | 214 |
|  |  |  | R: CTTCATCCGCCTCAGAAGAC |  |
| *Ocp22996* | 22996 | 520 | F: TGTCATTCTCTAAGACTGAGCATATAA | 190 |
|  |  |  | R: GCAGGACTTGTGGCTCTTTC |  |
| *Ocp3161* | 3161 | 600 | F: AGCAGACATTGCACTGGAGA | 186 |
|  |  |  | R: GGGAACCATTCCTCAACTCA |  |
| *Ocp9059* | 9059 | 265 | F: AGAGCCAGATTGAGGACGTG | 165 |
|  |  |  | R: GGCCGAGCAGTATTAACGAA |  |
| *Ocp20131* | 20131 | 535 | F: GAAGCTCTGGTTGGCTTTTG | 175 |
|  |  |  | R: TCTGCTTCGGGAAGTTCAGT |  |
| *Ocp5210* | 5210 | 602 | F: TGTAAATAGTTTGTTAATGCAGAGTCA | 160 |
|  |  |  | R: TGTACAAGAATAAAACAGTCAAGTCT |  |
| *Ocp37363* | 37363 | 208 | F: ACAAGATCTGCAAGGGCATT | 170 |
|  |  |  | R: GCAAAGCTAAACGTGGCAAT |  |
| *Ocp14033* | 14033 | 205 | F: CCTAGGGGTAGCTCACCTGTT | 180 |
|  |  |  | R: ACCCACTCCTGGGAAATTCA |  |
| *Ocp7546* | 7546 | 332 | F: GAGATGGTGACCAAGCACCT | 204 |
|  |  |  | R: TTTGTGGCCATGAAAGTTGT |  |
| *Ocp3475* | 3475 | 802 | F: CCCAGAGAAGAAGCAACCTG | 159 |
|  |  |  | R: CCATGCATGCTCTCTGTCAT |  |
| *Ocp2120* | 2120 | 839 | F: GATGCAGCAACTGAAGCAAA | 184 |
|  |  |  | R: GGCCAGACTATCCCACCTAA |  |
| *Ocp1135* | 1135 | 363 | F: GCTCAAAATGCCCTGAGGTA | 143 |
|  |  |  | R: GGGCACTCAGGAACGTGTAT |  |
| *Ocp687* | 687 | 295 | F: CAGAAGGAGGAGGCAGAGAA | 143 |
|  |  |  | R: TTCATCTCGCTGAAGAGAAGC |  |
| *Ocp3279* | 3279 | 533 | F: GACAAACCAGCTTTCCCATAA | 176 |
|  |  |  | R: ATGGGAAACATTCCACCATC |  |
| *Ocp2940* | 2940 | 928 | F: CTTTTCCTGGAGGCCAAAC | 212 |
|  |  |  | R: TTCTGCAGAGCCCTCTATGG |  |
| *Ocp31442* | 31442 | 457 | F: GGCCTAATTCTGACAGTCCTCA | 175 |
|  |  |  | R: GGGAGGGTTACGCCTGTATT |  |
| *Ocp28110* | 28110 | 713 | F: CTTTCTTTGCCCCCTTTCTT | 171 |
|  |  |  | R: TCATAGTCCAATGGAGAAGGAG |  |
| *Ocp4142* | 4142 | 194 | F: ATGACGTCCTAAGCCTGGAG | 174 |
|  |  |  | R: TCACAGTGGCCACATCTCTC |  |
| *Ocp3019* | 3019 | 139 | F: TGATTCAGAGTAGGCAGAAGTTCA | 175 |
|  |  |  | R: GTTAGCGAGAAGGCGTGAAG |  |
| *Ocp6361* | 6361 | 1397 | F: GCCAGTGGAACACTTGCAT | 155 |
|  |  |  | R: TGGAAAATCTGAAGAAAGGTTTAAG |  |
| *Ocp5637* | 5637 | 139 | F: GGTGACCTTGCATTCTCTGC | 184 |
|  |  |  | R: CTGTTCCTTCCCAGTGTGCT |  |
| *Ocp27622* | 27622 | 268 | F: GTTGGGGGAAGATGATGAAA | 150 |
|  |  |  | R: GAAATTTGGCATAGTTCAGTTCC |  |
| *Ocp2135* | 2135 | 136 | F: TGTCAAATGAACTTTCCAAACG | 183 |
|  |  |  | R: TTGGTAAACAATAATTGGCAACA |  |
| *Ocp10964* | 10964 | 400 | F: GACGACATGGACAAGGGTCT | 204 |
|  |  |  | R: CAGGGACAGATGCTTCCAAT |  |
| *Ocp1984* | 1984 | 280 | F: TTCTTCAAAGAGGCACAGAGC | 194 |
|  |  |  | R: AACATCAGAACAGAATTAAGTGTTCAA |  |
| *Ocp34088* | 34088 | 121 | F: CCCCTCCCCAAGTATCTGTC | 181 |
|  |  |  | R: GTTCTGGAAGCCTGTGGAAT |  |
| *Ocp26780* | 26780 | 290 | F: TGACTGTGTTGGTTCATCAGTG | 204 |
|  |  |  | R: TCTGTTCGAAACTAAAGGACACA |  |
| *Ocp951* | 951 | 520 | F: GAGCCTGACAATTTCTTCCTG | 182 |
|  |  |  | R: AACGCTTGGAGAAACAGAGG |  |
| *Ocp102174* | 102174 | 295 | F: AGGGGTTCCCTCATCTCTTC | 180 |
|  |  |  | R: CCACACTGCTGTTTGACGAC |  |
| *Ocp102175* | 102175 | 124 | F: TTGTATTTGCATGGCACACC | 166 |
|  |  |  | R: CAGCATCTTGAGGAGGAGGA |  |
| *Ocp15508* | 15508 | 64 | F: CAGCCCGTCAGTAAGAGAGG | 150 |
|  |  |  | R: GGCAGACCCCTTGAGACATA |  |
| *Ocp1829*^a^ | 1829 | - | F: GGTAGTGGTTGCAGTGTTAAGG | 447 |
|  |  |  | R: ACAAACGCCTGAGCCCTACT |  |
| *Ocp24554*^a^ | 24554 | - | F: ACCCTATCCGCCTTAACAAT | 810 |
|  |  |  | R: GGATTGGGCTAGTTTGTTGG |  |

^a^Primers used for ND5 Sanger sequencing.
