# Supplementary material for: Novel genomic resources for a climate change sensitive mammal: characterization of the American pika transcriptome
Source: BMC Genomics. 2013 May 10;14:311. doi: 10.1186/1471-2164-14-311 (PMC3662648; doi:10.1186/1471-2164-14-311)
Supplement: Additional file 6 — Sample collection. Word document (.doc) describing the samples used to generate each cDNA library. [file 1471-2164-14-311-S6.doc]

| Elevation | Sample number | Coordinates | Sex | RBCM Cat # |
| --- | --- | --- | --- | --- |
| High | A01/11 | N52.51716 | Male | 20919 |
|  |  | W125.82661 |  |  |
|  | A02/11 | N52.51716 | Female | 20920 |
|  |  | W125.82661 |  |  |
|  | A03/11 | N52.51732 | Male | 20924 |
|  |  | W125.82724 |  |  |
|  |  |  |  |  |
| Low | D01/11 | N52.41092 | Male | 20921 |
|  |  | W125.92439 |  |  |
|  | D02/11 | N52.41092 | Female | 20922 |
|  |  | W125.92439 |  |  |
|  | D03/11 | N52.41104 | Female | 20923 |
|  |  | W125.92475 |  |  |
